# Molecular Mechanisms and Potential New Therapeutic Drugs for Liver Fibrosis

**DOI:** 10.3389/fphar.2022.787748

**Published:** 2022-02-11

**Authors:** Fa-Da Wang, Jing Zhou, En-Qiang Chen

**Affiliations:** Center of Infectious Diseases, West China Hospital, Sichuan University, Chengdu, China

**Keywords:** liver fibrosis, hepatic stellate cells, cytokines, extracellular matrix, traditional Chinese medicine

## Abstract

Liver fibrosis is the pathological process of excessive extracellular matrix deposition after liver injury and is a precursor to cirrhosis, hepatocellular carcinoma (HCC). It is essentially a wound healing response to liver tissue damage. Numerous studies have shown that hepatic stellate cells play a critical role in this process, with various cells, cytokines, and signaling pathways engaged. Currently, the treatment targeting etiology is considered the most effective measure to prevent and treat liver fibrosis, but reversal fibrosis by elimination of the causative agent often occurs too slowly or too rarely to avoid life-threatening complications, especially in advanced fibrosis. Liver transplantation is the only treatment option in the end-stage, leaving us with an urgent need for new therapies. An in-depth understanding of the mechanisms of liver fibrosis could identify new targets for the treatment. Most of the drugs targeting critical cells and cytokines in the pathogenesis of liver fibrosis are still in pre-clinical trials and there are hardly any definitive anti-fibrotic chemical or biological drugs available for clinical use. In this review, we will summarize the pathogenesis of liver fibrosis, focusing on the role of key cells, associated mechanisms, and signaling pathways, and summarize various therapeutic measures or drugs that have been trialed in clinical practice or are in the research stage.

## Introduction

Hepatic fibrosis is a universal pathological process that occurs in various types of chronic liver disease, including viral hepatitis, alcoholic hepatitis, fatty liver disease, nonalcoholic fatty liver disease (NAFLD), wilson’s disease, and cholangitis. When hepatocytes are damaged, the release of signals such as reactive oxygen species (ROS) and intercellular interactions lead to the differentiation of HSCs towards myofibroblasts, and the latter is the primary source of the extracellular matrix (ECM) (Casini et al., 1997; Novo et al., 2009; Ghatak et al., 2011; Mederacke et al., 2013). Damaged hepatocytes also activate inflammatory cells such as macrophages and lymphocytes to generate multiple types of cytokines, including transforming growth factor-β (TGF-β) and platelet-derived growth factor (PDGF). These cytokines would result in dysregulation of ECM degradation and synthesis, leading to the development of liver fibrosis (Luedde et al., 2014; Seki and Schwabe, 2015). Suppose the injury persists and therapeutic interventions are not taken in time, the liver parenchyma will gradually be replaced by scar tissue formed by excessive ECM, leading to the loss of standard structure and the formation of cirrhosis. Additionally, the risk of hepatocellular carcinoma (HCC) and serious complications such as gastrointestinal bleeding increased.

Over the past decades, we have made some important progress in the mechanism study of liver fibrosis, but the complex pathogenesis of liver fibrosis poses certain difficulties for the development of anti-hepatic fibrosis drugs. Many therapeutic interventions are effective in experimental models, but their efficacy and safety in humans are unknown and cannot be applied in the clinic for the time being. Though still lack of specific anti-fibrosis agents in clinical, numerous studies have shown that the etiological treatment of primary liver disease is effective and even partially reversible for liver fibrosis (Dienstag et al., 2003; Czaja and Carpenter, 2004; Schiff et al., 2008; Chang et al., 2010). In addition, it is worth mentioning that traditional Chinese medicine (TCM) has a beneficial effect on anti-fibrosis (Chen et al., 2015). In this review, we will summarize the various therapeutic measures or drugs that have been trialed in clinical practice or are in the research stage.

## Overview of the Mechanisms of Liver Fibrosis

**FIGURE 1:**
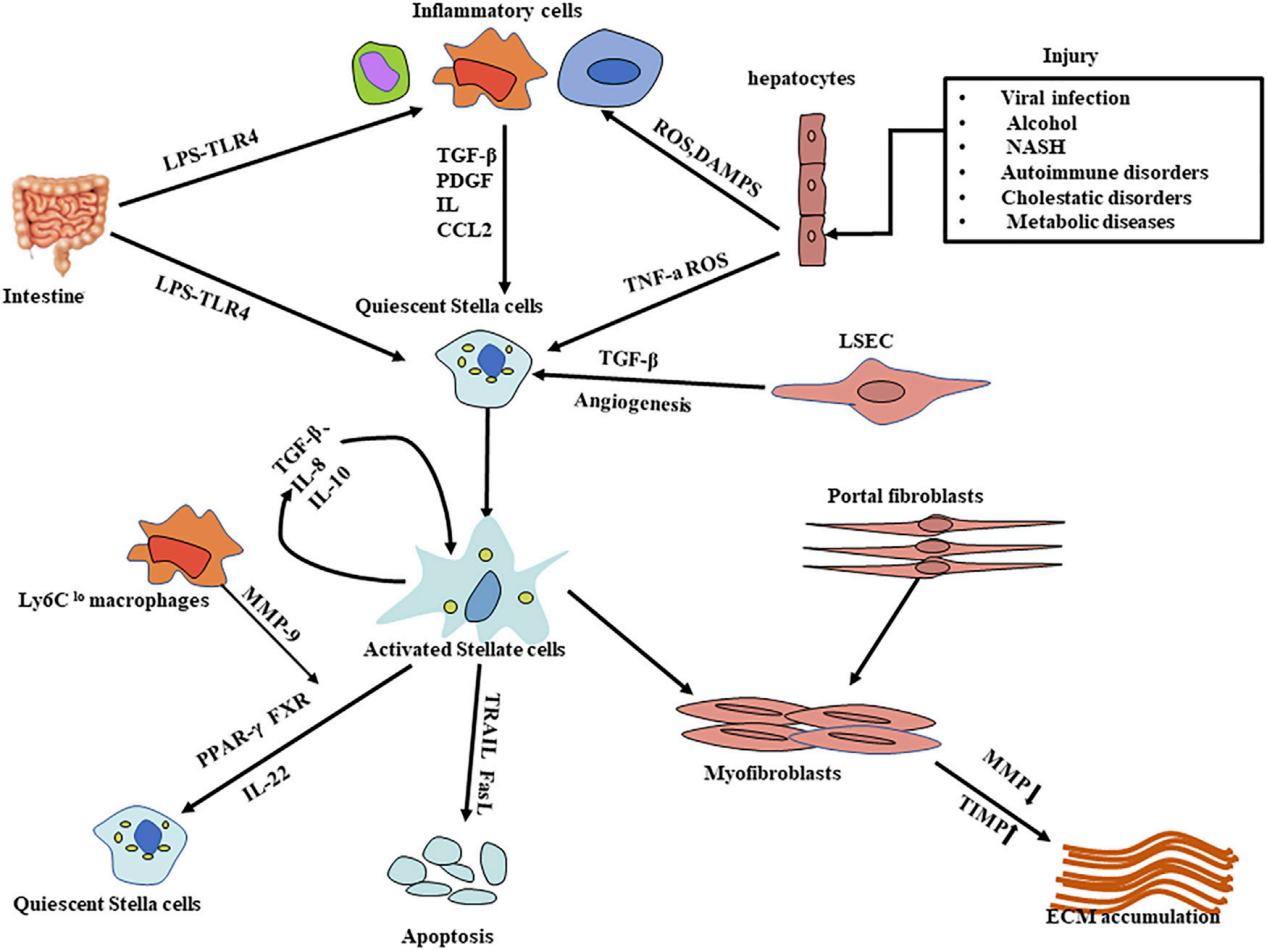
Mechanisms of liver fibrosis. Liver injury is caused by a variety of stimuli that result in hepatocyte damage and the release of substances such as ROS; in response to persistent hepatocyte injury, HSCs and macrophages (including Kupffer cells) are activated, activated myofibroblasts increase and excessive ECM is produced, leading to the progression of liver fibrosis. The activation of hepatic stellate cells is a key step in the process of liver fibrosis. Many influential factors regulating HSC activation, proliferation, function, and survival have emerged as important therapeutic targets; likewise, protection of hepatocytes from damage and degradation of excessive ECM deposition provide therapeutic options. HSCs: Hepatic stellate cells CCL2:C-C chemokine ligands types 2; LPS: Lipopolysaccharide LSEC： Liver sinusoidal endothelial cells TIMP: inhibitors of matrix metalloproteinase; MMP: matrix metalloproteinase; DAMPS:damage-associated molecular patterns; ECM: extracellular matrix; ROS: reactive oxygen species.

Liver fibrosis is caused by an excessive accumulation of scar tissue, accompanied by angiogenesis (Lin et al., 2021), which ultimately leads to changes in the architecture of the liver. The mechanisms of liver fibrosis can be generalized as follows, multiple stimuli (such as toxins, viruses, cholestasis, hypoxia, and insulin resistance, etc.) attack the liver cells and induce the formation of reactive oxygen species (such as hydrogen peroxide, hydroxyl radicals, and aldehyde end products, etc.), which in turn cause hepatocyte damage, apoptosis, steatosis, and immune cell infiltration, especially kupffer cells (KCs) (Wehr et al., 2013; Pradere et al., 2013). At the same time, sinusoidal endothelial cells experience the loss of fenestrae, known as capillarization of the sinusoids (Marrone et al., 2016). Chronic damage to hepatocytes is the initiator of the fibrotic cascade, it induces the production of pro-fibrotic cytokines/growth factors (e.g., TNF-a, IL-6, TGF-β, and PDGF) indirectly through interactions with hepatic macrophages and natural killer (NK) cells. Meanwhile, it directly activates primary response cells (e.g., hepatic stellate cells) through the release of cellular contents, ultimately leading to the activation of HSCs and the fibrotic network and excessive deposition of ECM (Figure 1) (Canbay et al., 2002; Elpek, 2014). Based on the pathogenesis, we can regress liver fibrosis by protecting hepatocytes, inhibiting the activation of hepatic stellate cells, and fibrotic scar evolution.

The ECM is a complex network of macromolecular substances that can regulate various physiological functions such as cell growth, proliferation, migration, differentiation, adhesion, metabolism, damage repair, and tissue remodeling through various signaling systems. In the normal liver, it is a highly dynamic substrate that maintains an exact balance between synthesis and degradation (Theocharis et al., 2016; Villesen et al., 2020). However, in chronic liver disease, the balance is disturbed due to the involvement of multiple cells and cytokines, leading to a greater synthesis than degradation. But most of these changes can be reversed if the liver injury is transient (Hernandez-Gea and Friedman, 2011). The process of liver fibrosis is complicated, involving both hepatic parenchymal and non-parenchymal cells as well as immune cells, and the main functions of different cells and cytokines in liver fibrosis are described in detail below.

## Key Cell Types in Liver Fibrosis

### Hepatic Stellate Cells and Myofibroblasts

In normal liver, HSCs exhibit a quiescent state, whose physiological functions are related to fat storage and the metabolism of vitamin A. Another function of the quiescent HSC is to secrete adequate amounts of ECM proteins such as type III collagen, type IV collagen, and laminin. Besides, HSC secretes a variety of degradative enzymes called matrix metalloproteinases (MMPs), such as MMP-1, which promote the degradation of ECM. HSC also produces tissue inhibitors of matrix metalloproteinases (TIMPs), such as the TIMP-1 and TIMP-2. The TIMP1 can prevent ECM degradation by blocking MMPs and can inhibit HSC apoptosis (Carloni et al., 1996; Roeb et al., 1997; Benyon and Arthur, 2001; Geerts, 2001; Yoneda et al., 2016). The highly regulated interaction between MMPs and TIMPs is responsible for the renewal of the liver matrix and the maintenance of homeostasis and healthy liver architectures *in vivo* (Murphy et al., 2002). When the liver injury occurs, numerous key cells and inflammatory mediators are involved, including inflammatory stimuli, fibrogenic cytokines TGF-β, ROS, produced by activating macrophages, platelets, and products of damaged hepatocytes drive HSC activation. Quiescent HSCs become activated and TIMP-1 expression is upregulated, which is an essential and central step of liver fibrogenesis. The activated HSCs can not only transform into myofibroblasts and secrete enough ECM, but also secret cytokines such as TGF-β to maintain a constant state of activation, ultimately resulting in the deposition of mature collagen fibers in the space of the Disse and leading to the formation of scars (Tsuchida and Friedman, 2017).

In past, our understanding of HSCs has been dominated by their crucial role in liver fibrosis, thus generating anti-fibrotic strategies that target this cell. As research has progressed and understanding of the role of HSCs in disease has increased, we have found that HSCs have a role in promoting liver cell regeneration (Yang et al., 2008), which may be achieved mainly through the following mechanisms, secretion of cytokines that promote liver cell proliferation, promote the migration of stem cells to the liver, and promote the epithelial transformation of mesenchymal cells into hepatocytes. Therefore, we need to consider their role in liver regeneration when targeting HSCs in liver fibrosis (Ge et al., 2020).

Myofibroblasts (MFs) are key cells in fibrotic diseases, including lung, kidney, and liver disease (Friedman et al., 2013). It is the major cell that produces ECM in the process of liver fibrosis, such as collagen I and III. The origin of myofibroblasts has been controversial, but experiments and data now demonstrate that the main sources are HSCs and portal myofibroblasts. Following an injury to liver tissue, myofibroblasts are transformed from activated HSCs in response to a large number of cytokines and inflammatory cells. The overproduced cytokines can continue to act on myofibroblasts to keep them activated, which in turn produces large aggregates of ECM. In biliary disease, the main source of myofibroblasts is portal myofibroblasts (Iwaisako et al., 2014; Wells et al., 2015). Besides, animal experiments have shown that HSCs and myofibroblasts can be converted from mesothelial cells via mesothelial-mesenchymal transition after liver injury (Li et al., 2013).

### Hepatocytes

Hepatocytes make up 80% of the total cell population and volume of the human liver, and under physiological conditions perform a variety of functions such as detoxification, secretion of bile, proteins, and lipids (Schulze et al., 2019). It is also a primary target for toxic substances that attack the liver. Hepatocyte death is an important initial event in all liver diseases. Dead hepatocytes release intracellular compounds called damage-associated molecular patterns (DAMPs) that signal to surround hepatic stellate cells and Kuffer’s cells and therefore play an important role in the development of fibrosis and inflammation (An et al., 2020; Gaul et al., 2021). Therefore, protecting hepatocytes from damage is an important therapeutic intervention.

### Inflammatory Cells

Inflammation is a fundamental characteristic of chronic liver disease, cell death is typically the precipitating event. The release of signals such as reactive oxygen species (ROS) from the damaged cells can activate the inflammatory cells, including macrophages, lymphocytes, and NK cells etc (Jaeschke, 2011). Among them, hepatic macrophages (Kupffer cells) play major roles and are known as regulators in the process of liver fibrosis (Wynn and Barron, 2010). KCs are an essential component of the innate immune mononuclear phagocytic system and play critical functions in homeostasis, and act as first responders following liver injury. In response to tissue damage, numerous Ly-6Chi macrophages are recruited to the liver, releasing cytokines and attracting NK cells and other immune cells (Karlmark et al., 2009; Reid et al., 2016). These macrophages could induce the transdifferentiation of HSCs into collagen-producing myofibroblasts by secreting TGF-β1 and PDGF. Dendritic cells (DCs) increase fibrosis regression, mainly through the production of MMP-9 (Jiao et al., 2012). NK cells directly kill target cells and are capable of producing a variety of cytokines that play various roles in liver injury, fibrosis, and hepatocarcinogenesis, activated NKT cells have a role in killing activated HSCs (Radaeva et al., 2006). However, in chronic liver disease, NKT cells have a pro-inflammatory function, recruit neutrophils and myeloid cells, and promote the activation of hepatic stellate, leading to hepatocyte necrosis, fibrosis, and even HCC (Jin et al., 2011; Wolf et al., 2014).

Activated inflammatory cells are the primary source of cytokines, such as C-C chemokine ligands types 2 and 5 (CCL2 and CCL5), IL, TGF-β1, PDGF, etc. The role of inflammatory cells is double-sided, which could promote the regression of liver fibrosis and accelerate the deterioration of fibrosis. For example, hepatic macrophages can not only relieve inflammation and fibrosis by degrading the ECM and releasing anti-inflammatory cytokines but also promote liver fibrosis by activating HSCs (Duffield et al., 2005; Tacke and Zimmermann, 2014). The Ly6Chi macrophages can differentiate into restorative Ly6Clo macrophages to engulf cell debris and secrete MMP-9 and MMP-1/MMP-2 to promote scar regression (Ramachandran et al., 2012).

### Liver Sinusoidal Endothelial Cells

In normal liver tissue, liver sinusoidal endothelial cells (LSECs) have characteristics of vasodilatory, anti-inflammatory, anti-thrombotic, anti-angiogenic, anti-fibrotic, and regeneration-promoting effects (Ding et al., 2010), so LSECs are considered to be the gatekeepers of hepatic homeostasis. At the same time, LSECs are the main source of endothelium-derived nitric oxide (NO), which keeps HSCs in a resting state. In the presence of liver injury, LSECs become capillarized, which can not only reduce the production of vasodilators (such as NO, cyclooxygenase, and prostaglandin I2 [PGI2]) but also increase the production of vasoconstrictors (endothelin 1, thromboxane A2, angiotensin II). This imbalance not only alters the phenotype of LSECs but also contributes to the activation of HSCs and promotes inflammation and liver fibrosis (Deleve et al., 2008; Xie et al., 2012; Poisson et al., 2017). It also secretes TGF-β, PDGF or activates signaling pathways such as Wntβcatenin, which can activate HSCs in a paracrine and autocrine manner. Due to the unique properties of LSECs, selective LSEC-targeted therapy appears to be an attractive strategy for the treatment of liver fibrosis (Gracia-Sancho et al., 2021).

## Molecular Signaling Pathways Involved in Liver Fibrogenesis

### TGF-β Signaling and Platelet-Derived Growth Factor Signaling

Transforming growth factors (TGF) have the function of regulating the growth and development of various cells, which are essential for the homeostasis of tissues and organs. In the liver, they are mainly produced by HSCs, LSECs, KCs, and DCs as well as NKT cells, and can act on themselves or other cells through autocrine or paracrine secretion (Schon and Weiskirchen, 2014). The functions of TGF-β vary between different types and stages of liver disease. During liver fibrosis, TGF-β is up-regulated, the main function of TGF-β is to activate HSCs, which are considered to be the main pro-fibrotic factor in the process of liver fibrosis. It also enhances the expression of TIMPs and directly promotes the synthesis of interstitial fibrillar collagens (García-Trevijano et al., 1999; Hellerbrand et al., 1999; Dewidar et al., 2019). Due to its important function in liver fibrosis, blocking the signaling pathway of TGF-β is now a potential target for the treatment of liver fibrosis (Guo et al., 2012).

The platelet-derived growth factor (PDGF) is a member of the family of growth factors whose biological functions include angiogenesis, regulation of cell proliferation and survival, cell migration, and stimulation of the synthesis of major components of the connective tissue matrix (Heldin and Westermark, 1999). In the context of liver disease, the expression of PDGF and its receptors have now been shown to be significantly upregulated (Pinzani et al., 1996). It has been regarded as the most effective growth factor for HSC proliferation in hepatic fibrosis, and the receptors of PDGF have become a new promising direction in the treatment of liver fibrosis (Pinzani et al., 1989; Borkham-Kamphorst et al., 2007).

### Inflammatory Cytokines Pathways

The progression and regression of liver fibrosis are regulated by a complex signaling pathway consisting of cytokines, growth factors, and chemokines. IL-6, TNF-a, Interleukins, PDGF, and TGF-β are the key pro-inflammatory and profibrogenic cytokines that drive liver fibrosis. Interleukins (ILs) are important immunomodulatory cytokines. During liver injury, it is produced by various cell types and exerts pro-inflammatory (such as IL-13, IL-17, and IL-33) as well as anti-inflammatory effects (such as IL-22 and IL-10) in hepatic cells (Hu et al., 2016; Xu et al., 2016; Liu et al., 2019). For instance, An animal study has shown that the IL-6/gp130 pathway plays a protective role for non-parenchymal hepatocytes in the progression of fibrosis (Streetz et al., 2003). But a recent study has demonstrated that IL-6 can induce differentiation of HSCs towards myofibroblast via MAPK and JAK/STAT signaling pathways (Kagan et al., 2017). In addition, IL-22 has been shown to attenuate liver fibrosis by binding to cell receptors, attenuating the activation of HSCs and down-regulating levels of inflammatory cytokines (Lu et al., 2015). This suggests that a strategy using blocking pro-inflammatory interleukins or inducing anti-inflammatory interleukin production to treat liver fibrosis can be effective.

Tumour necrosis factor (TNF) and related receptor pathways can activate apoptosis in hepatocytes *via* the caspases pathway and exert anti-apoptotic effects *via* the NF-κB pathway (Osawa et al., 2018). TNF also plays a vital role in the activation of HSCs and the synthesis of ECM (Osawa et al., 2013). TNF reduces apoptosis of activated rat HSCs through upregulation of the anti-apoptotic factor NF-κB. However, the effects of TNF-α on HSCs and fibrosis are multiple. In animal experiments, it has shown an anti-fibrotic effect by reducing glutathione and inhibiting the secretion of pro-collagen α1 (Hernandez-Munoz et al., 1997; Varela-Rey et al., 2007).

### Toll-Like Receptors in Liver Fibrosis

The liver is exposed to venous blood from the small and large intestines through the portal vein. Due to this unique blood supply system, the liver is easily exposed to bacterial products that are transferred from the lumen of the intestine *via* the portal vein. The small and large intestines are rich in flora. In healthy organisms, due to the barrier effect of the intestinal mucosa, only a small amount of translocated bacterial products reach the liver, and the liver immune system tolerates these bacterial products to avoid harmful reactions. After the injury to the liver or the intestinal mucosa, the flora becomes disturbed, and intestinal bacteria can translocate to the liver. Its metabolites such as lipopolysaccharide (LPS) can conjugate with functional toll-like receptor 4 (TLR4) to activate reactive cells such as HSCs and Kupffer cells, and also enhance the activity of transforming growth factors thus leading to the development of liver fibrosis (Seki et al., 2007; Schnabl and Brenner, 2014). In the liver, both hepatocytes and non-parenchymal cells (NPCs) have expressed TLR4. Compared to other organs, healthy livers have a low level of TLR4. However, damaged livers increase the expression of TLR4 and its co-receptors, thus making TLR4 signaling-mediated inflammatory responses more sensitive (Kitazawa et al., 2008; Guo and Friedman, 2010).

## Potentially Effective Treatments for Liver Fibrosis

Recently, it has been shown that fibrosis can reverse after the removal of pathogenic conditions. Although no drugs are currently approved for the treatment of liver fibrosis, some treatment modalities have shown effectiveness in patients, such as antiviral therapy for patients with viral hepatitis, zinc for wilson’s disease, phlebotomy for hemochromatosis, alcohol withdrawal for alcoholic liver disease and ursodeoxycholic acid (UDCA) in the treatment of primary biliary cholangitis (Powell and Klatskin, 1968; Marcellini et al., 2005; Takahashi et al., 2014; Bardou-Jacquet et al., 2020; Ye et al., 2020). glucocorticoids, vitamin E, and angiotensin receptor antagonists have gradually been shown to have antifibrotic effects as well. In addition, TCM appears to have an increasingly prominent role in the treatment of liver fibrosis and its efficacy is promising (Fujiwara et al., 2010; He et al., 2013), but more clinical trials are needed to confirm its effectiveness. In the following, we will describe these potential treatments in detail below (Table 1).

**TABLE 1:** Targets and main mechanisms of some of existing anti-fibrotic drugs and novel therapeutic approaches.

Agent	Anti-fibrotic target	Mechanism	Refs
Etiological treatment	Etiology	Removal of causative factors	(Powell and Klatskin, 1968; Marcellini et al., 2005; Takahashi et al., 2014; Rockey, 2016; Lens et al., 2017; Tada et al., 2018; Bardou-Jacquet et al., 2020; Ye et al., 2020)
Glucocorticoids	HSC lymphocytes	Reducing the transmission of transforming growth factors	(Bolkenius et al., 2004; Czaja, 2014)
		Weakening the activity of hepatic stellate cells	
		Inhibiting the proliferation of lymphocytes	
Curcumin	Inflammation cell and inflammation response	Anti-inflammatory and antioxidant effects	(Zhang et al., 2014; Zhao et al., 2018; Kong et al., 2020)
		Blocking the epithelial-mesenchymal transition of hepatocytes	
		Inhibiting the activation of Kuffer cells	
		Inhibiting NF-κB upregulation and reducing sinusoidal angiogenesis	
YCHD	TGF-*β* and RAS system	Reduction of RAS pathway components and down-regulation of TGF expression	Wu et al. (2015)
XCHT	Nrf2	Inhibition of hepatic stellate cell activation	Li et al. (2017)
Baicalein	PDGF receptors	Inhibit the activation and value-added of hepatic stellate cells by down-regulating PDGF receptors	Sun et al. (2010)
FFBJ	HSC	Inhibition of hepatic stellate cell proliferation and activation, as well as limiting the expression of TGF-β1 and PDGF	Yang et al. (2016)
GW570	HSC	A PPARγ receptor agonism, simulating PPARγ mediated gene transcription	Yang et al. (2010)
Obeticholic acid		An FXR agonist, FXR expressed in hepatic stellate cells has an anti-fibrotic effect	Mudaliar et al. (2013)
Pioglitazone		A PPARγ receptor agonism	Musso et al. (2017)
Nilotinib		Inhibition of TK, TK activation transforms HSC into an activated state	(Ma et al., 2017) (Shaker et al., 2013)
Sorafenib			
β-aminopropionitrile	ECM	Inhibits LOX, LOX-mediated cross-linking of collagen limits MMP degradation of ECM	Georges et al. (2007)
CVC	Cytokines	Dual antagonist of the CCR type 2 and 5	Friedman et al. (2018)
TG101348		JAK2 receptor antagonist	Akcora et al. (2019)
E5564	TLR4	Inhibitors of TLR4	(Fort et al., 2005; Kitazawa et al., 2009; Takashima et al., 2009)
P13			
CRX526			
vitamin E	ROS	Antioxidant effects	(Sanyal et al., 2010; Bril et al., 2019; Miyazawa et al., 2019)
losartan/candesartan	AT1 receptor	Angiotensin II may exert its pro-fibrotic effects, Blocking or attenuating the role of Angiotensin II.	(Colmenero et al., 2009; Kim et al., 2012)
RNA interference	target genes	Downregulation of genes of critical cytokines in activated HSCs	(Chen et al., 2008; Cong et al., 2013; Zhou and Yang, 2014; Jiménez Calvente et al., 2015)
MiRNAs	mRNAs	Trigger the degradation of target mRNAs about liver fibrosis	(Chen et al., 2018; Wang et al., 2019)
MSC	Inflammation	Modulation of the hepatic immune response	
	HSC	Secretion of trophic cytokines to reduce hepatocyte apoptosis	(Eom et al., 2015)
	MMPs/TIMP-1	Antioxidant effects	
		Inhibition of HSC appreciation	
		Increased expression of MMPs	
		Reduced expression of TIMP-1	

Abbreviations: PPAR, proliferator-activated receptor; HSCs, Hepatic stellate cells; FXR, farnesoid X receptor; ECM, extracellular matrix; ROS, reactive oxygen species; TK, Tyrosine kinase; JAK, Janus kinase; TLR, Toll-like receptor; P13, a peptide called P13; YCHD, Yinchenhao Decoction; XCHT, Xiaochaihutang; FFBJ, Fufang Biejia Ruangan Tablets; MSC, Mesenchymal stem cell; PDGF, platelet growth factor RAS, renin-angiotensin system.

### Antiviral Therapy

Among all the factors that contribute to chronic liver disease, hepatitis virus infection is the most common, primarily hepatitis B and C. Chronic hepatitis B virus infection is a worldwide public health problem, with approximately 250 million people chronically infected and at high risk of developing cirrhosis and liver cancer. When liver cells are infected with the virus, cellular damage induces an inflammatory response, at the same time the virus itself can directly induce activation of the immune system, leading to activation of HSCs and progress to liver fibrosis. Clearing hepatitis viruses or inhibiting hepatitis virus replication is the most effective way to reduce liver cell damage. With the advent of antiviral drugs, we have now made considerable progress in the fight against the hepatitis B and C virus. Through effective antiviral therapy, most liver fibrosis can be reversed, and liver cirrhosis and its related complications can be reduced (Rockey, 2016; Lens et al., 2017; Tada et al., 2018).

### Drugs Targeting Inflammation

Glucocorticoids have immunomodulatory and anti-inflammatory effects. As we mentioned earlier, the inflammatory response and immune cells play critical roles in the process of liver fibrosis. Thus glucocorticoids may have some therapeutic effects in liver fibrosis. It has been shown that glucocorticoids can reduce liver fibrosis by reducing the transmission of transforming growth factors, weakening the activity of HSCs, and inhibiting the proliferate of lymphocytes, but the efficacy of glucocorticoids differently in different diseases (Bolkenius et al., 2004; Czaja, 2014). Glucocorticoids or immunosuppressive agents are the most significant treatment options for chronic autoimmune liver disease, and liver fibrosis can be reversed with adequate management (Valera et al., 2011). Early glucocorticoid treatment is effective for prognosis in hepatitis and liver failure due to viral hepatitis B (Fujiwara et al., 2010; He et al., 2013). Nevertheless, the use of corticosteroids for alcohol-related acute liver failure or slow-onset acute liver failure is still controversial in clinical practice, although the AASLD and EASL guidelines recommend treatment with corticosteroids. Studies have shown that the use of glucocorticoids improves short-term survival but does not significantly improve long-term prognosis and carries risks such as infection (Thursz et al., 2015; Sersté et al., 2018; Gustot and Jalan, 2019). Similarly, the use of glucocorticoids to treat a drug-induced liver injury is also in dispute (Andrade et al., 2019). Consequently, there is still a need for extensive trials and data to evaluate the safety and efficacy of glucocorticoids in liver disease.

### Traditional Chinese Medicine With Multiple Effects on Liver Fibrosis

Notably, there is growing evidence that TCM is effective in the prevention and treatment of liver fibrosis (Pan et al., 2020). TCM can suppress liver fibrosis activity through different mechanisms, including inhibition of cytokine production and suppression of HSCs activation, as well as regulating the progression of liver fibrosis through other molecular mechanisms (Shan et al., 2019). Turmeric is an herb that grows in Asia and has been widely used as a spice in food and for therapeutic applications. In China, it is also an ingredient in TCM and recently its extract curcumin has received much attention. Curcumin has been widely used in anti-fibrotic models due to its anti-inflammatory and antioxidant effects. It has been shown to alleviate liver fibrosis by blocking the epithelial-mesenchymal transition of hepatocytes through the regulation of oxidative stress and autophagy (Cai et al., 2018; Cai et al., 2019), and to weaken the role of Ly6C^hi^ cells in liver fibrosis by inhibiting the activation of Kuffer cells, thereby reducing the secretion of chemokines (Cai et al., 2018; Cai et al., 2019). In addition, it also has the effect of inhibiting NF-κB upregulation and reducing sinusoidal angiogenesis (Cai et al., 2018; Cai et al., 2019). More research is underway on the mechanism of curcumin against liver fibrosis.

YCHD (Yinchenhao Decoction) is a traditional Chinese herbal formulation used to treat liver fibrosis and has been experimentally shown to have multiple active ingredients targeting various targets of liver fibrosis (Cai et al., 2018; Cai et al., 2019). Recent studies have indicated RAS system in liver fibrosis/cirrhosis may exert pro-fibrotic effects, and the antifibrotic effects of the YCHD fibrosis effect may be related to the reduction of RAS pathway components and down-regulation of TGF expression (Bataller et al., 2000; Yoshiji et al., 2007). XCHT (Xiaochaihutang) is a water decoction traditionally used in china for the treatment of liver diseases, and the mechanism for its anti-fibrosis is not completely clear. Nrf2 is an important redox-sensitive transcription factor *in vivo*, which can promote cell survival, as well as maintain the redox state of cells. Animal experiments have shown that XCHT is an effective drug for the treatment of liver fibrosis, and its therapeutic effect is mainly through upregulation of the Nrf2 pathway thus resulting in the inhibition of HSCs activation (Bataller et al., 2000; Yoshiji et al., 2007). In addition, baicalein, the main component of XCHT, can inhibit the activation and proliferation of HSCs by down-regulating PDGF receptors, thus exerting an anti-fibrotic effect (Bataller et al., 2000; Yoshiji et al., 2007). It has been reported that ETV combined with FFBJ (Fufang Biejia Ruangan Tablets) showed significant anti-fibrotic effects in CHB patients. The mechanism of action may be related to the inhibition of HSCs proliferation and activation, as well as limiting the expression of TGF-β1 and PDGF (Bataller et al., 2000; Yoshiji et al., 2007).

Similar TCMs also include Huangqi Decoction Dahuang Zhechong Pills, Fuzheng Huayu Formula, Anluo Huaxian Pills (Li, 2020), etc. Although TCM has been used for thousands of years, its clinical effectiveness in liver fibrosis needs further evaluation due to the lack of rigorous randomized controlled trials.

### Vitamin E and Renin-Angiotensin System Inhibitor

Vitamin E, angiotensin-converting enzyme inhibitors (ACE-I), and angiotensin II type 1 (AT1) receptor blockers have recently drawn attention to the treatment of liver fibrosis. Vitamin E is an important nutrient with antioxidant effects, it can inhibit the production of singlet free radicals, oxygen, lipid hydroperoxides, and lipid radicals. Since products such as free radicals play an important role in the development of liver fibrosis, it may be a potential option for the treatment of liver fibrosis. However, in patients with non-alcoholic liver disease, vitamin E could not significantly reduce the severity of fibrosis (Sanyal et al., 2010; Bril et al., 2019; Miyazawa et al., 2019).

Recent studies have also shown that the production of angiotensin II type 1 receptor is increased in activated HSCs and enhanced the activity of the renin-angiotensin system (RAS) in liver fibrosis/cirrhosis. Angiotensin II may exert its pro-fibrotic effects through increased oxidative stress, activation and proliferation of HSCs, upregulation of TGF-β and TIMP1, and accelerated deposition of collagen (Bataller et al., 2000; Yoshiji et al., 2007). Based on these mechanisms, ACE-I and AT1 receptor blockers are potential treatment options for liver fibrosis. A clinical study evaluating the efficacy of angiotensin II receptor blocker (ARB) losartan in patients with HCV showed that it can reduce inflammation and decrease fibrosis gene expression (Colmenero et al., 2009).In patients with chronic alcoholic liver diseases, the combination of UDCA and ARB candesartan improved patients’ fibrosis scores compared to UDCA treatment alone (Kim et al., 2012). However, as no clear effects have been shown in other clinical trials, further studies are needed to demonstrate the benefit of RAS antagonists in liver fibrosis.

## Candidate Therapeutic Targets in Clinical Trials

There are no directly effective anti-fibrotic drugs in clinical practice and most of them are still in clinical trials and in the research stage. Although etiological treatment has proven to be effective, some etiologies cannot be eliminated. In addition, even with effective etiological treatment, reversal of advanced liver fibrosis cannot completely avoid complications such as gastrointestinal bleeding and HCC, so we urgently need direct anti-fibrotic drugs.

The main pathogenesis of liver fibrosis can be summarized as follows: Following the chronic injury to hepatocytes, the multitude of cellular and cytokine interactions lead to the activation of key cells such as HSCs and MFs, which in turn leads to the overproduction of ECM and the development of liver fibrosis (Parola and Pinzani, 2019; Kisseleva and Brenner, 2021). Based on the pathogenesis of liver fibrosis, the drugs we are exploring focus on the following aspects: protecting hepatocytes from damage, inhibiting cytokine activity and cell proliferation, promoting apoptosis of key cells, reducing ECM synthesis, and promoting its degradation. Many relevant drugs are already in clinical trials, including FXR agonists, PPAR agonists, and TAK agonists, which inhibit hepatic stellate cell activation and promote ECM degradation, TLR4 receptor antagonists, and novel therapeutic approaches such as miRNA and mesenchymal stem cell therapy. Below we describe in detail the novel treatments and drugs according to the different targets (Table 1).

### Inhibition and Reversal of the Activation of Hepatic Stellate Cells

Because of its important role in liver fibrosis, HSCs become a major target for anti-fibrotic drugs (Elpek, 2014). Reversing liver fibrosis by converting activated HSCs to a quiescent state or promoting their apoptosis is our main goal. Experimental models of fibrosis consistently demonstrate that elimination of activated HSCs by apoptosis or other pathways can lead to regression of fibrosis (Iredale et al., 1998; Troeger et al., 2012). Recent studies have shown that activated HSCs can be transformed into non-fibrotic cells by transcriptional reprogramming, such as ectopic expression of GATA4, FOXA3, HNF1a, and HNF4a *in vivo* (Song et al., 2016). Besides, cellular senescence may be an anti-fibrotic strategy. Expression of nuclear receptors PPAR and FXR in HSCs suppress HSCs activation, as studies have shown that HSC senescence can be invoked by PPARγ, FXR agonist, such as GW570 (Krützfeldt et al., 2005; Janssen et al., 2013; Thakral and Ghoshal, 2015), pioglitazone (Krützfeldt et al., 2005; Janssen et al., 2013; Thakral and Ghoshal, 2015), obeticholic acid (Krützfeldt et al., 2005; Janssen et al., 2013; Thakral and Ghoshal, 2015), thereby alleviating liver fibrosis degree. TK (Tyrosine kinase) is expressed in HSCs and its activation transforms them into an activated state, thus inhibition of TK may be a potential target for the treatment of liver fibrosis. Sorafenib has been used as a treatment for patients with HCC, where complications of cirrhosis (such as portal hypertension) have been reduced, and its anti-fibrotic activity has been confirmed in numerous trials (Ma et al., 2017). Nilotinib triggers apoptosis and autophagic cell death via inhibition of histone deacetylase in activated HSC cells (Krützfeldt et al., 2005; Janssen et al., 2013; Thakral and Ghoshal, 2015). Unfortunately, most of these drugs are in animal studies and are not yet available for clinical use, their safety and efficacy deserve to be evaluated.

### Reduction of Fibrotic Scar Evolution

The removal of the excess ECM is one of our goals for treating liver fibrosis. Collagen is the most abundant ECM protein in liver fibrosis. Specific inhibition of type 1 collagen fibrils synthesis has now been achieved in animals by miRNA, and this miRNA leads to a significant reduction in collagen 1 synthesis in fibrosis models (Jiménez Calvente et al., 2015). Besides, LOX is a copper-dependent amine oxidase (Perepelyuk et al., 2013), and LOX-mediated cross-linking of collagen limits MMP degradation of ECM. The β-aminopropionitrile inhibits LOX, reduces liver stiffness, decreases the number of fibroblasts, and attenuates cell injury-induced liver fibrosis (Georges et al., 2007). However, the clinical trial did not demonstrate significant efficacy (Loomba et al., 2018). Similar to LOX, transglutaminase (TGs) forms a covalent isopeptide bond by covalently linking a glutamine residue of one protein chain to a lysine residue of another protein chain. Intercross-linking of TGs can promote liver fibrosis, and therefore invoking specific inhibitors of TGs could be a potential target for the treatment of liver fibrosis (Van Herck et al., 2010).

### Drugs Targeting Cytokines and Signaling Pathways

Cytokines are involved in the entire process of liver fibrosis, blocking their signaling pathways, and receptors may inhibit the production of the ECM and accelerate its degradation. Cenicriviroc (CVC), an oral dual antagonist of the CCR type 2 and 5, has been shown to have antifibrotic effects in animal studies. A clinical trial has shown amelioration of liver fibrosis in patients with nonalcoholic steatohepatitis (NASH) after 1 year of treatment with CVC (Krützfeldt et al., 2005; Janssen et al., 2013; Thakral and Ghoshal, 2015), and further clinical trials on CVC are currently underway, and we hope that it will become an anti-fibrotic option in the future. The Janus kinases (JAK) signaling pathway plays an important role in the pathogenesis of hepatic fibrosis and can be activated by a variety of cytokines such as IL. Studies have shown that the use of the JAK2 receptor antagonist TG101348 can reduce hepatic fibrosis in animal models (Krützfeldt et al., 2005; Janssen et al., 2013; Thakral and Ghoshal, 2015). However, cytokine function is also important for maintaining the immune response, tissue repair, etc. Long-term targeting of these cytokines is challenging due to the severe adverse effects. A great deal of research has been done on cytokine antagonism (Gressner and Weiskirchen, 2006).

### Drugs Targeting TLR4

As we mentioned earlier that intestinal microbiota is closely associated with the development of liver fibrosis. the main mechanism by which liver fibrosis occurs, in this case, is the combination of the bacterial metabolites LPS and TLR4, further activating key cells in the liver fibrosis process. Therefore, inhibition of TLR4-related intracellular signaling may be effective in reducing TLR4-mediated inflammation and inhibiting liver fibrosis (Beutler, 2004). It was shown that a peptide called P13, which was previously shown to be a potent inhibitor of TLR signaling *in vitro*. Using this peptide to treat mice effectively inhibited LPS-induced inflammatory mediator production and significantly limited liver damage, enhancing survival in a mouse model of inflammation (Tsung et al., 2007). Several small-molecule inhibitors of TLR4 are currently being tested, including lipid A mimetics, e.g., E5564 and CRX526 (Fort et al., 2005; Kitazawa et al., 2009; Takashima et al., 2009), soluble fusion proteins with extracellular structural domains. However, these are still in animal studies and may become targets for anti-fibrotic drugs in the future.

### siRNA and miRNA in Liver Fibrosis

Liver fibrosis is highly related to activated HSCs, and the activation of HSCs is regulated by a variety of cytokines. Downregulation of these cytokines in activated HSCs using RNA interference (RNAi) is a promising strategy for reversing liver fibrosis. RNA interference is a new technique that uses small interfering RNAs (siRNAs) of 21–23 nucleotides to specifically knock out target genes, and this new technique is based on the high specificity of siRNAs and their ability to downregulate genes associated with liver fibrosis (Kim and Rossi, 2007). Many therapies for siRNA are currently in clinical trials to translocate siRNA into HSC or other hepatic parenchymal cells, for example, lipid nanoparticles containing HSP47 siRNA for the treatment of liver fibrosis (Kulkarni et al., 2018). The main mechanism of siRNA action is to cause homologous degradation of the targeted mRNA (Aagaard and Rossi, 2007). It has been shown that siRNA can address liver fibrosis by regulating collagen expression in HSC (Krützfeldt et al., 2005; Janssen et al., 2013; Thakral and Ghoshal, 2015). Meanwhile, it has been reported that direct knockdown of TGF-β expression using siRNA can exert antifibrotic effects in a rat model (Krützfeldt et al., 2005; Janssen et al., 2013; Thakral and Ghoshal, 2015). Similarly, the use of PDGF siRNA suppressed the advancement of liver fibrosis in mice (Krützfeldt et al., 2005; Janssen et al., 2013; Thakral and Ghoshal, 2015). Besides, MMP2-specific siRNA and TIMP-specific siRNAs also exert an anti-fibrotic effect on the liver (Krützfeldt et al., 2005; Janssen et al., 2013; Thakral and Ghoshal, 2015).

MiRNAs are endogenous small non-coding RNAs that can post-transcriptionally regulate the expression of mRNAs and ultimately trigger the degradation of target mRNAs. miRNAs are associated with a variety of liver diseases, including liver fibrosis, and therefore miRNAs are an alternative treatment for liver fibrosis. It has been found that miRNAs can be both up and down-regulated during liver fibrosis. Up-regulated miRNA can be reverted by anti-miRNA oligonucleotides, and miRNA masking (Krützfeldt et al., 2005; Janssen et al., 2013; Thakral and Ghoshal, 2015), unlike the upregulated miRNA, some downregulation of miRNA inhibiting liver fibrosis has been found (Chen et al., 2018; Wang et al., 2019), down-regulated MiRNA can be restored by MiRNA mimics or plasmids expressing miRNA (Cheng and Mahato, 2011). Similar to siRNAs, the biggest challenge for miRNAs is to overcome degradation and targeted transport in the blood. To date, there are no clinical trials on MiRNA about the treatment of liver fibrosis. A lot of effort has been spent on siRNA with MiRNA and viral and non-viral transport systems have been developed, which also still face significant challenges. There are already anti-fibrotic siRNAs in clinical trials, and in the future siRNA with miRNA may become a novel treatment for liver disease.

### Mesenchymal Stem Cell Therapy for Liver Fibrosis

Recently, MSC therapy has been regarded as an effective alternative for the treatment of liver disease. MSCs possess the ability to self-renew and differentiate into many types of cells, and differentiation of MSCs into hepatocytes is the prospect of liver regeneration (Hu et al., 2020). The main mechanisms of the anti-fibrotic effects of MSCs can be generalized as follows, modulation of the hepatic immune response, secretion of trophic cytokines to reduce hepatocyte apoptosis, antioxidant effects, inhibition of HSC proliferation, and increased expression of MMPs or reduced expression of TIMP-1 (Hernandez-Munoz et al., 1997; Varela-Rey et al., 2007). Mesenchymal stem cells are now widely used in clinical and preclinical studies of liver fibrosis, Jang and others showed the beneficial effects of autologous bone marrow MSC transplantation for the treatment of alcoholic cirrhosis (Jang et al., 2014), Kharaziha et al. (2009) showed that liver function improved in patients with cirrhosis after injected autologous mesenchymal stem cells. However, due to their multi-differentiation potential, MSCs can differentiate into myofibroblasts rather than hepatocytes (Baertschiger et al., 2009; di Bonzo et al., 2008). Besides, another risk of MSC transplantation is that they are susceptible to malignant transformation and promote the growth of existing tumors (Zhu et al., 2006). MSCs have the potential to differentiate into hepatocytes, immunomodulatory properties, and the ability to secrete trophic cytokines, making them a potential treatment for liver disease. however, with both their fibrotic potential and their ability to promote the growth of pre-existing tumor cells, MSC therapy needs to be evaluated further.

## Conclusion

Recently, with our greater understanding of the mechanisms of liver fibrosis, a plethora of therapeutic strategies have been generated. but the treatment of liver fibrosis remains a difficult clinical problem that we face today and etiological treatment is currently recognized as the most effective anti-fibrotic approach. Multiple interactions between ECM, hepatic stellate, endothelial cells, and immune cells have been demonstrated during liver fibrosis, but the central event in fibrosis is the activation of HSCs. Due to multiple cells and cytokines being involved in the progression of liver fibrosis, it is crucial for us to fully understand the biology of critical cells such as HSCs, myofibroblasts, and macrophages, including their activation and inactivation, to facilitate the development of specific targeted drugs. In addition, the inflammatory response is one of the fundamental features of liver fibrosis, so controlling liver inflammation and inflammatory cells is also a viable strategy for treating liver fibrosis. Besides, novel therapies targeting intestinal microecology, mRNA, and mesenchymal stem cells are also becoming available for clinical trials, and several drugs have been successful in regressing liver fibrosis in experimental models.

A growing number of potential drugs are in phase II and III trials, and we expect that some of these drugs may soon be approved for use in patients. These new drugs target multiple pathways in the pathogenesis of chronic liver disease, but the mechanisms of liver fibrosis are complex. With certain cells having a dual role in the development and regression of liver fibrosis, and targeted therapies may have some side effects. Therefore we must understand the mechanisms more clearly so that we can establish scientific treatments that are safe and effective in achieving long-term results. In the future, a better understanding of the molecular mechanisms involved in the regression of liver fibrosis may provide new preventive and therapeutic strategies for patients with fibrosis and even cirrhosis.
